# Overexpression of quality control proteins reduces prion conversion in prion-infected cells

**DOI:** 10.1074/jbc.RA118.002754

**Published:** 2018-08-28

**Authors:** Simrika Thapa, Basant Abdulrahman, Dalia H. Abdelaziz, Li Lu, Manel Ben Aissa, Hermann M. Schatzl

**Affiliations:** From the ‡ Calgary Prion Research Unit, University of Calgary, Calgary, Alberta T2N 4Z6, Canada,; the § Department of Comparative Biology and Experimental Medicine, Faculty of Veterinary Medicine, University of Calgary, Calgary, Alberta T2N 4Z6, Canada,; the ¶ Department of Biochemistry and Molecular Biology, Faculty of Pharmacy, Helwan University, 11795 Cairo, Egypt, and; the ‖ Departments of Veterinary Sciences and of Molecular Biology, University of Wyoming, Laramie, Wyoming 82071

**Keywords:** prion, prion disease, ER quality control, endoplasmic reticulum stress (ER stress), infection, lentivirus, bovine spongiform encephalopathy, Creutzfeldt–Jakob disease, neurodegeneration, scrapie

## Abstract

Prion diseases are fatal infectious neurodegenerative disorders in humans and other animals and are caused by misfolding of the cellular prion protein (PrP^C^) into the pathological isoform PrP^Sc^. These diseases have the potential to transmit within or between species, including zoonotic transmission to humans. Elucidating the molecular and cellular mechanisms underlying prion propagation and transmission is therefore critical for developing molecular strategies for disease intervention. We have shown previously that impaired quality control mechanisms directly influence prion propagation. In this study, we manipulated cellular quality control pathways *in vitro* by stably and transiently overexpressing selected quality control folding (ERp57) and cargo (VIP36) proteins and investigated the effects of this overexpression on prion propagation. We found that ERp57 or VIP36 overexpression in persistently prion-infected neuroblastoma cells significantly reduces the amount of PrP^Sc^ in immunoblots and prion-seeding activity in the real-time quaking-induced conversion (RT-QuIC) assay. Using different cell lines infected with various prion strains confirmed that this effect is not cell type– or prion strain–specific. Moreover, *de novo* prion infection revealed that the overexpression significantly reduced newly formed PrP^Sc^ in acutely infected cells. ERp57-overexpressing cells significantly overcame endoplasmic reticulum stress, as revealed by expression of lower levels of the stress markers BiP and CHOP, accompanied by a decrease in PrP aggregates. Furthermore, application of ERp57-expressing lentiviruses prolonged the survival of prion-infected mice. Taken together, improved cellular quality control via ERp57 or VIP36 overexpression impairs prion propagation and could be utilized as a potential therapeutic strategy.

## Introduction

Prion diseases are transmissible spongiform encephalopathies characterized by distinctive spongiform appearance and loss of neurons in the brain. Transmissible spongiform encephalopathies include Creutzfeldt–Jakob disease in humans, scrapie in sheep, bovine spongiform encephalopathy in cattle, and chronic wasting disease in cervids. These diseases are caused by accumulation of the misfolded infectious isoform (PrP^Sc^) of the normal cellular prion protein (PrP^C^) in the brain and other tissues (1–3). Unlike α-helix–rich PrP^C^, PrP^Sc^ is rich in β-sheets, insoluble in detergent, and partially protease-resistant, making the two isoforms distinguishable by immunoblotting after proteinase K (PK) ^2^ digestion (4–7). PrP^Sc^ accumulation in the brain leads to formation of aggregates that impair the normal physiology of the brain, leading to neurodegeneration. Prion diseases are unique among neurodegenerative diseases, as they have the potential to be transmitted between and within species, including zoonotic transmission from animals to humans (8–10). There is currently no treatment available for these fatal neurodegenerative diseases.

The endoplasmic reticulum (ER) plays an important role in the cell biology of PrP^C^, including its folding, posttranslational modification, translocation to the secretory pathway, and quality control (11). ER quality control of proteins allows the transportation of only correctly folded proteins out of the ER to the target cellular compartments, ensures the degradation of misfolded proteins, and helps to maintain homeostasis in the secretory pathway. The accumulation of misfolded proteins in the ER (12) alters its physiological function and perturbs ER homeostasis, leading to ER stress. It is important for cells to avoid such ER stress, which might lead to accumulation of misfolded protein deposits and cellular toxicity (13). The unfolded protein response (UPR) is a series of coordinated signaling events initiated and regulated by cells to cope with ER stress. It results in downstream effects leading to attenuation of protein translation and up-regulation of chaperones and other quality control molecules to cope with misfolded proteins and enhance protein degradation (14). Some of the components of ER quality control are chaperones and cargo receptors. Chaperones help with correct folding of proteins, prevent protein aggregation, and allow proper translocation of proteins. For example, BiP helps with folding of proteins, is involved in protein translocation, and regulates the UPR (15–17). Folding enzymes such as calnexin, calreticulin, and thiol-disulfide oxidoreductase (ERp57) are also involved in folding and quality control (18). Cargo receptors such as ERGIC-53 and VIP36 ensure that only correctly folded proteins exit the ER to the Golgi apparatus (19, 20). The latter helps with retrograde transport and brings misfolded proteins from the Golgi back to the ER.

Several reports have suggested that incorrectly folded forms of PrP^C^ and PrP^Sc^ accumulate during ER stress in prion disease models (21–24). Moreover, it has been shown in *in vitro* and *in vivo* models that prion infection resulted in cells undergoing ER stress, which further facilitates the formation of misfolded PrP^C^ and increased prion conversion (22, 24–26). Previous studies in our laboratory have also demonstrated a direct influence of impairment in quality control mechanisms on prion conversion, and overexpression of quality control proteins such as ERGIC-53 and EDEM-3 reduced prion conversion (24). Another group showed that overexpression of BiP modulated prion propagation *in vitro* and in animal models (27). Thus, the manipulation of cellular quality control mechanisms could be a potential strategy for interfering in prion conversion by helping only correctly folded PrP^C^ to reach the plasma membrane, which is less prone to prion conversion. Additionally, it has been reported that ERp57 has a protective effect *in vitro* against prion toxicity and regulates the expression and maturation of PrP^C^ in cells (28, 29).

In this study, we investigated the role of overexpression of proteins involved in folding (ERp57) and secretory protein cargo transport (VIP36) on prion conversion. In persistently prion-infected cells, we found a significant reduction of PrP^Sc^ following overexpression. We used both stable and transient overexpression systems, different cell types, and different prion strains to assess the effect on prion propagation. Moreover, when ERp57- or VIP36-overexpressing noninfected cells were infected with prions, we found that the overexpressing cells were less susceptible to prion infection. Additionally, ERp57-overexpressing cells showed reduced susceptibility to induction of ER stress. These results provide strong evidence for the role of quality control in prion infection. Together with our preliminary *in vivo* data, this suggests that ERp57 and VIP36 could be promising targets against prion infection. Thus, manipulation of the protein quality control mechanisms could lead to reduced PrP^Sc^ conversion.

## Results

### Stable overexpression of ERp57 or VIP36 reduces PrP^Sc^ in prion-infected neuroblastoma cells

**Figure 1.**
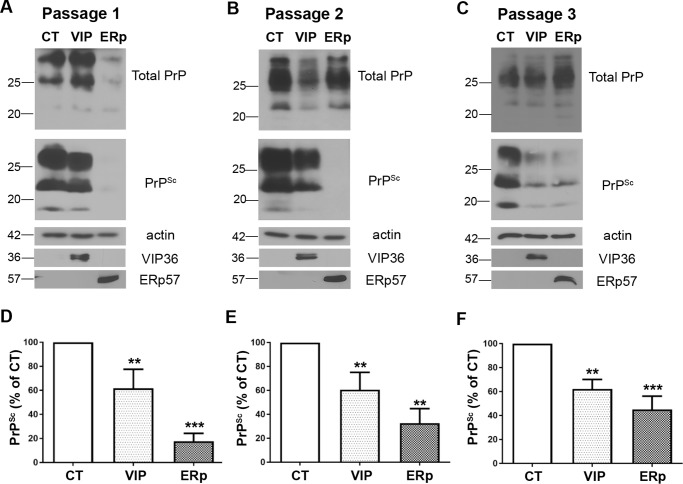
**Stable overexpression of VIP36 or ERp57 reduces PrP^Sc^ in prion-infected cells.** ScN2a-22L cells were stably transduced with recombinant lentiviruses to overexpress either VIP36 (*VIP*) or ERp57 (*ERp*) or control GFP virus (*CT*). Cells were then passaged and lysed at passages 1, 2, and 3. Cell lysates were subjected to PK digestion (+PK) or not (−PK) to distinguish total PrP and PrP^Sc^. The PrP amount was analyzed by immunoblot using anti-PrP mAb 4H11. Overexpression of VIP36 and ERp57 was detected by staining with anti-HA and anti-myc antibodies for exogenous ERp57 and VIP36, respectively, as shown in the *bottom panels*, indicating successful transduction. Actin was used as a loading control. *A–C*, representative immunoblots showing the reduction of PrP^Sc^ in the first passage (*A*), second passage (*B*), and third passage (*C*). *D*, densitometric analysis showing the amount of PrP^Sc^ in the first passage normalized by the actin amount and shown as a percentage of control using ImageJ. *E*, densitometric analysis for the second passage. *F*, densitometric analysis for the third passage. Data represent mean ± S.E. (*n* = 5–8). **, *p* ≤ 0.01; ***, *p* ≤ 0.001.

To investigate the role of ERp57 and VIP36 in prion replication, we stably overexpressed ERp57 or VIP36 in N2a cells persistently infected with mouse-adapted scrapie prion strain 22L (ScN2a-22L) using a lentiviral gene integration technique. ScN2a-22L cells were transduced with lentiviruses that integrated genes encoding ERp57 (HA-tagged) or VIP36 (myc-tagged) into the host genome, allowing stable overexpression of genes. Transduced cells were selected using puromycin. When nonvirally transduced cells were subjected to puromycin selection as a control, all cells were susceptible to puromycin treatment. As lentiviral transduction resulted in expression of GFP along with the target gene (dual promoter construct), successful transduction and selection of cells were confirmed by investigating GFP autofluorescence with fluorescence microscopy and target protein expression with Western blotting. The transduced cells were passaged. At each passage, cells were lysed, and the lysates were subjected to PK digestion and immunoblotting. Upon overexpression of ERp57, we found a significant reduction of PrP^Sc^ in the first passage compared with control cells transduced with mock virus (Fig. 1, *A* and *D*). This reduction of PrP^Sc^ was also present in passages 2 and 3 (Fig. 1, *B*, *C*, *E*, and *F*). Moreover, VIP36 overexpression significantly reduced PrP^Sc^, as shown in Fig. 1. Exogenous overexpression of ERp57, tagged with HA, and VIP36, tagged with c-myc, was confirmed using anti-tag antibodies in immunoblot analysis.

**Figure 2.**
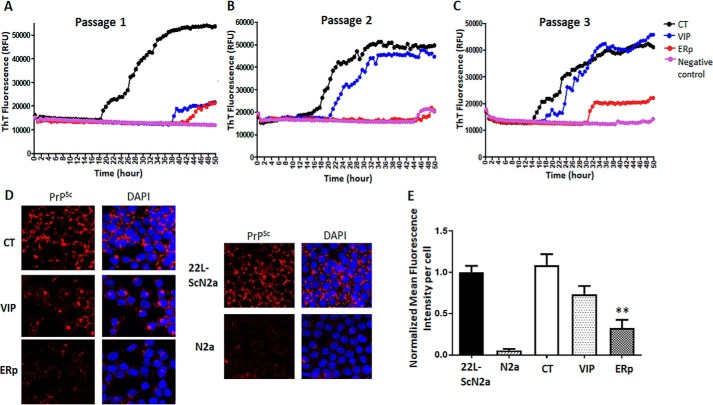
**RT-QuIC analysis shows reduced prion seeding activity in VIP36- or ERp57-overexpressing prion-infected cells.** ScN2a-22L cells were transduced with lentiviruses to stably overexpress VIP36 (*VIP*) or ERp57 (*ERp*) or control GFP virus (*CT*). Cells were then passaged and lysed in passages 1, 2, and 3. In each passage, cell lysates were collected, and RT-QuIC analysis was performed. The *y* axis shows relative ThT fluorescence units (*RFUs*) and the *x* axis time in hours. N2a cell lysate was used as a negative control. The seeding activity at 10^−2^ dilution was analyzed to compare the groups. *A–C*, RT-QuIC analysis for passage 1 (*A*), passage 2 (*B*), and passage 3 (*C*). *D*, ScN2a-22L cells stably overexpressing VIP36 or ERp57 were subjected to 6 m guanidine treatment, and PrP^Sc^ was stained with anti-PrP mAb 4H11 (*left panels*). *Right panels*, 4′,6-diamidino-2-phenylindole (*DAPI*) merge. N2a cells were used as negative control and nontransduced and control-transduced cells as positive control. Confocal microscopy revealed the PrP^Sc^ amount in cells. *E*, the mean fluorescence intensity per cell was quantified to analyze the amount of PrP^Sc^ for each group using ImageJ. **, *p* ≤ 0.01.

Moreover, we tested cells for changes in prion seeding activity using real-time quaking-induced conversion (RT-QuIC) assay. In this test, recombinant PrP^C^ substrate is converted into ThT-binding aggregates in the presence of prion seeds. Mouse rPrP was used as substrate, and cell lysates in dilutions from 10^−1^ to 10^−4^ served as seed in RT-QuIC, as described previously (30). We found reduced prion seeding activity in cell lysates of ERp57- or VIP36-overexpressing cells compared with control cells (10^−2^ dilution shown) (Fig. 2, *A* and *B*). At passage 3, we found less seeding activity of cell lysates from ERp57-overexpressing cells (Fig. 2*C*).

To further validate the immunoblot results, we used immunofluorescence microscopy for semiquantitative detection of PrP^Sc^ in cells. Immunofluorescence analysis involving pretreatment with guanidine salts for epitope retrieval is widely used for specific detection of PrP^Sc^ (31). Cells in passage 3 were subjected to this immunofluorescence analysis after treatment with 6 m guanidine hydrochloride for PrP^Sc^ using anti-PrP mAb 4H11 and Cy3 goat anti-mouse as primary and secondary antibodies, respectively. The mean immunofluorescence intensity per cell in PrP^Sc^ immunostaining was compared among transduction groups. Noninfected cells were used as a negative control and to eliminate background staining. Confocal microscopy analysis confirmed the reduction of PrP^Sc^ in ERp57- or VIP36-overexpressing cells (Fig. 2, *D* and *E***)**. Taken together, these data show that stable overexpression of ERp57 or VIP36 in persistently prion-infected cells reduces PrP^Sc^ and prion conversion activity.

### Transient overexpression of ERp57 or VIP36 decreases PrP^Sc^ accumulation in cultured cells infected with different prion strains

**Figure 3.**
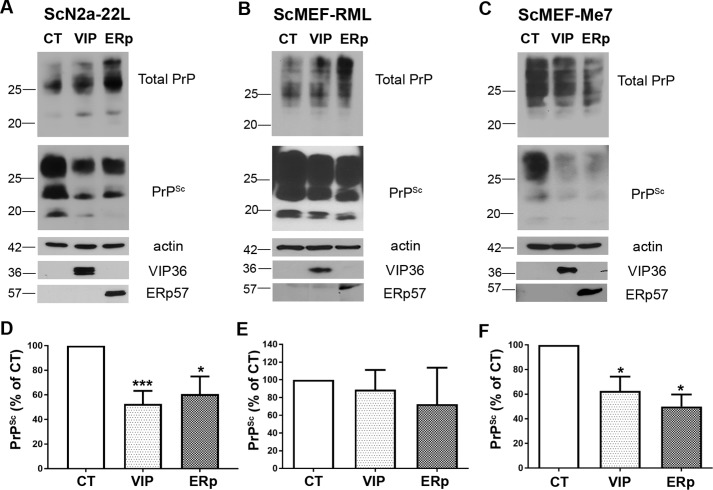
**Transient overexpression of VIP36 and ERp57 decreases PrP^Sc^ in prion-infected cells.** Persistently prion-infected ScN2a-22L cells, ScMEF-RMLs, and ScMEF-Me7s were transfected twice with VIP36 (*VIP*) or ERp57 (*ERp*) or a control construct (*CT*) for 48 h each, and cells were lysed 96 h after the first transfection. The cell lysates were then subjected to PK digestion (or not, −PK), and the amount of total PrP (−PK) and PrP^Sc^ (+PK) was investigated using immunoblotting (anti-PrP mAb 4H11). *A–C*, representative immunoblots are shown for ScN2a-22L cells (*A*), ScMEF-RML cells (*B*), and ScMEF-Me7 cells (*C*). Actin was used as a loading control. Transfection was confirmed by detecting the overexpression of VIP36 and ERp57 using anti-HA and anti-myc antibodies for ERp57 and VIP36, respectively, as shown in the *bottom panels. D–F*, densitometric analysis showing the amount of PrP^Sc^ presented as percentage of control normalized by actin shown as mean ± S.E. in ScN2a-22L (*D*, *n* = 7), ScMEF-RML (*E*, *n* = 3), and ScMEF-Me7 (*F*, *n* = 4) cells. *, *p* ≤ 0.05; ***, *p* ≤ 0.001.

Next we wanted to investigate whether the inhibitory effect of overexpression of selected molecules on prion propagation is cell type– and prion strain–specific. We transiently transfected ScN2a-22L cells and mouse embryonic fibroblasts (MEFs) infected with RML or Me7 prions (ScMEF-RML and ScMEF-Me7, respectively) with plasmids expressing ERp57 or VIP36 using Lipofectamine LTX plus. Cells were consecutively transfected, with a 48-h interval, and lysed 96 h after the first transfection. Lysates were treated with proteinase K (+PK) and analyzed by immunoblot. These studies showed that ERp57 overexpression significantly decreased the PrP^Sc^ amounts in ScN2a-22L and ScMEF-Me7 cells (Fig. 3, *A*, *C*, *D*, and *F*). However, this effect was not significant in ScMEF-RML cells (Fig. 3, *B* and *E*). Similarly, VIP36 overexpression resulted in a significant reduction of PrP^Sc^ in the analyzed cell populations except in ScMEF-RML cells (Fig. 3, *B* and *E*). These data show that overexpression of ERp57 and VIP36 reduces PrP^Sc^ in various cell types infected with different prion strains.

### Stable and transient overexpression of ERp57 results in increased levels of PrP^C^ in neuroblastoma cells

**Figure 4.**
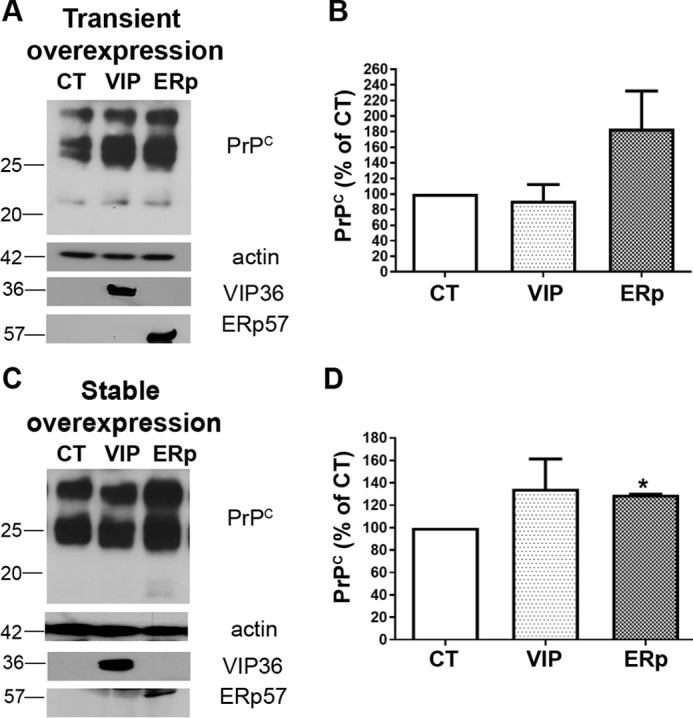
**ERp57 overexpression increases PrP^C^ expression in N2a cells.** *A*, N2a cells were transiently transfected with VIP36 (*VIP*) and ERp57 (*ERp*) or a control construct (*CT*) for 48 h. Cells were then lysed, and the postnuclear lysates were used for quantifying the amount of PrP^C^ by immunoblot analysis. Actin was used as a loading control, and transfection was confirmed with anti-HA and anti-myc antibodies for detection of tagged ERp57 and VIP36, respectively. *B*, densitometric analysis of PrP^C^ expression represented as percentage of control. Data represent mean ± S.E. (*n* = 5). *C* and *D*, N2a cells stably overexpressing VIP36 and ERp57 or control cells were lysed at passage 1, and immunoblotting was done for analyzing the amounts of PrP^C^ in postnuclear lysates as above. *D*, densitometric analysis (*n* = 3). *, *p* ≤ 0.05.

The previous results had shown that overexpression of the ER quality control proteins ERp57 or VIP36 prevented prion propagation *in vitro* and decreased PrP^Sc^ levels. One explanation for this is that such overexpression might modulate the levels of PrP^C^, which resides in the secretory pathway. PrP^C^ reduction, for example, would, in turn, impede PrP^Sc^ propagation. Indeed, previous studies have already shown that ERp57 plays a regulatory role in the expression of PrP^C^ and that ERp57 overexpression increases PrP^C^ levels (29). To investigate the role of ERp57 and VIP36 overexpression in PrP^C^, we transiently transfected N2a cells with plasmids encoding ERp57 or VIP36. 48 h after transfection, cells were lysed, and the lysates were subjected to immunoblot analysis. ERp57-overexpressing cells showed increased amounts of PrP^C^ compared with control cells (Fig. 4, *A* and *B*). Next, stably transduced N2a cells overexpressing ERp57 or VIP36 were analyzed. Again, we found that overexpression of ERp57 significantly increased the amount of PrP^C^ (Fig. 4, *C* and *D*). In contrast, VIP36 had little or no effect on PrP^C^ levels (Fig. 4). These data suggest that the reduction of PrP^Sc^ because of ERp57 or VIP36 overexpression was not due to a reduction in PrP^C^ levels. Moreover, quantitative PCR data suggested that PrP mRNA expression was unchanged in overexpressing cells compared with control cells (data not shown). It is conceivable that overexpression helped to generate PrP^C^ of good quality on the cell membrane, less prone to prion conversion, as described previously by us for overexpression of other quality control proteins (24). Taken together, these data clearly indicate that the decrease in PrP^Sc^ upon ERp57 or VIP36 overexpression in prion-infected cells is not due to a reduction of total levels of PrP^C^.

### ERp57 and VIP36 overexpression decreases the susceptibility of cells to prion infection

**Figure 5.**
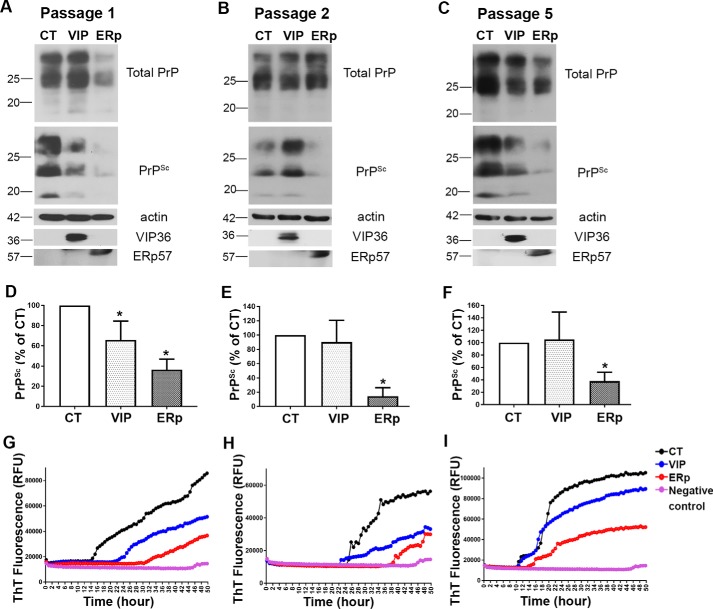
**ERp57 overexpression reduces *de novo* prion infection in cells (acute infection).** Uninfected N2a cells stably overexpressing VIP36 (*VIP*) and ERp57 (*ERp*) or control cells (*CT*) were infected with 1% brain homogenate of terminally ill 22L-infected mice. *A–C*, cells were passaged and lysed at passage 1 (*A*, at first splitting after infection), passage 2 (*B*), and passage 5 (*C*). The postnuclear lysates were subjected to PK digestion or not to assess newly formed PrP^Sc^ in each passage. Actin was used as a loading control, and expression of exogenous ERp57 and VIP36 was confirmed by utilizing anti-HA and anti-myc antibodies, respectively. *D–F*, densitometric analysis showing the amount of PrP^Sc^ normalized by the amount of β-actin and expressed as a percentage of control at passage 1 (*D*), passage 2 (*E*), and passage 5 (*F*) (*n* = 3–4). *G–I*, the RT-QuIC assay was done at passage 1 (*G*), passage 2 (*H*), and passage 5 (*I*) using the postnuclear lysates as seed. The seeding activity at 10^−2^ dilution was used for comparative analysis. The *y* axis shows relative ThT fluorescence units and the *x* axis time in hours. N2a cell lysate was used as a negative control. Note that VIP36-overexpressing cells showed a similar PrP^Sc^ signal as control cells in the immunoblot but lower seeding activity than the control in RT-QuIC over all passages. *, *p* ≤ 0.05.

The results above showed that overexpression of selected quality control proteins counteracted prion propagation in cells already infected with prions. This was in line with previous studies by us and others using overexpression of quality control proteins such as ERGIC-53, EDEM-3, and BiP in persistently prion-infected cells (24, 27). Next, we wanted to know whether this effect also exists in *de novo* prion infection. To investigate the impact of overexpression on the susceptibility of cells to prion infection, we overexpressed ERp57 or VIP36 in uninfected N2a cells and then infected cells with brain homogenate from terminally prion-sick mice. Aliquots of cells were lysed at each passage, and the lysates were subjected to immunoblot analysis for PrP (with or without PK) and to the RT-QuIC assay for testing seeding activity. Interestingly, we found that ERp57-overexpressing cells harbored less PrP^Sc^ in the immunoblot and less seeding activity in RT-QuIC compared with controls (Fig. 5). This finding was consistent until passage 5 (Fig. 5, *C* and *F*). Although VIP36-overexpressing cells showed a minor difference in the amount of PrP^Sc^ in the immunoblot in this situation compared with controls (Fig. 5, *A–C*), their prion seeding activity was reduced compared with controls in RT-QuIC analysis (Fig. 5, *G* and *H*). In summary, these results suggest that overexpression of ERp57 and VIP36 protects cells in *de novo* prion infection and reduces susceptibility to cellular prion infection.

### Overexpression of quality control proteins prevents ER stress–induced accumulation of PrP aggregates

**Figure 6.**
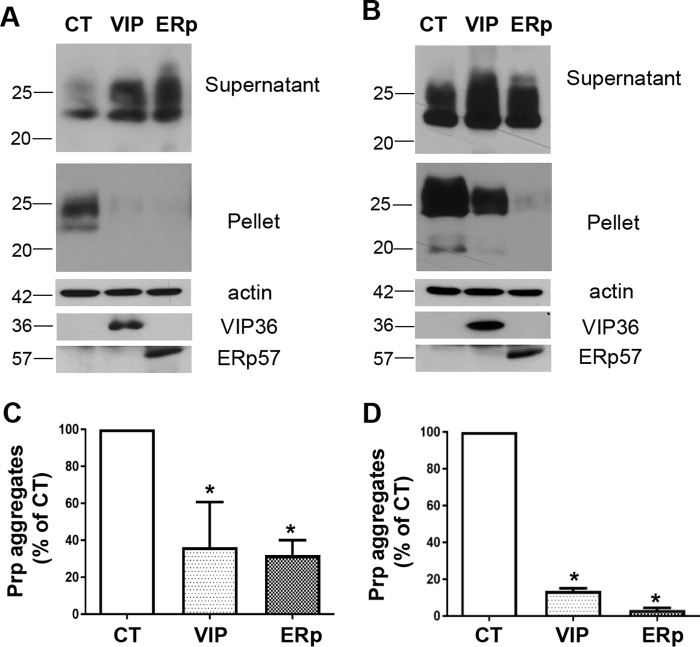
**ERp57 or VIP36 overexpression reduces PrP aggregates in cells.** *A* and *C*, N2a cells transiently overexpressing ERp57 (*ERp*) or VIP36 (*VIP*) or cells transfected with a control plasmid (*CT*) were treated with tunicamycin (2.5 μg/ml) 48 h after transfection for 16 h to induce ER stress in the cells. The cells were lysed and subjected to a detergent solubility assay. The postnuclear lysates were ultracentrifuged at 100,000 × *g* for 1 h at 4 °C in the presence of 1% sarcosyl. The supernatant and pellet fractions were tested in immunoblotting for PrP (pellet and supernatant), actin, and transgene expression (supernatant fraction only). *A*, representative immunoblot showing PrP distribution in pellet and supernatant fractions. *C*, densitometric analysis indicating PrP aggregates represented as percentage of control (*n* = 3). *B* and *D*, induction of ER stress in stably overexpressing N2a cells at passage 2 after transduction. Shown is a similar analysis as in *A*; tunicamycin treatment was for 16 h after 72 h of plating the cells. Representative immunoblots are shown in B, and densitometric analysis (*n* = 3) is depicted in *D*. **, *p* ≤ 0.01.

Overexpression of ERp57 or VIP36 was successful in reducing prion infection in persistently and newly prion-infected neuronal and nonneuronal cells. However, the molecular mechanisms underlying this process had to be determined. Our previous studies suggested that ER stress caused by accumulation of misfolded proteins results in deterioration of the quality of the PrP^C^ pool, PrP aggregate formation, and induced prion propagation in infected cells (24). Consequently, we wanted to investigate whether overexpression of ERp57 or VIP36, both components of the protein quality control pathway, can be beneficial in ER stress situations. We therefore transfected N2a cells with plasmids encoding ERp57, VIP36, or control constructs. After 48 h of transfection, the cells were treated with tunicamycin (2.5 μg/ml) for 16 h. Tunicamycin is an ER stress inducer that inhibits *N*-glycosylation during glycoprotein synthesis in the ER by blocking UDP-GlcNAc-dolichol phosphate GlcNAc-1-phosphate transferase (32). Cells were then lysed, and the postnuclear lysates were subjected to a detergent solubility assay as described previously (24). The supernatant and pellet fractions were analyzed for PrP amounts in an immunoblot. ERp57- or VIP36-overexpressing cells significantly overcame ER stress induced in the cells compared with control cells by expressing lower levels of aggregated PrP in the pellet fraction (Fig. 6, *A* and *C*). Similarly, we treated cells stably overexpressing ERp57 or VIP36 with tunicamycin for 16 h after 72 h of plating and then subjected postnuclear lysates to a detergent solubility assay. PrP aggregate formation in ERp57- and VIP36-overexpressing cells was significantly reduced in the pellet fraction compared with control cells (Fig. 6, *B* and *D*).

**Figure 7.**
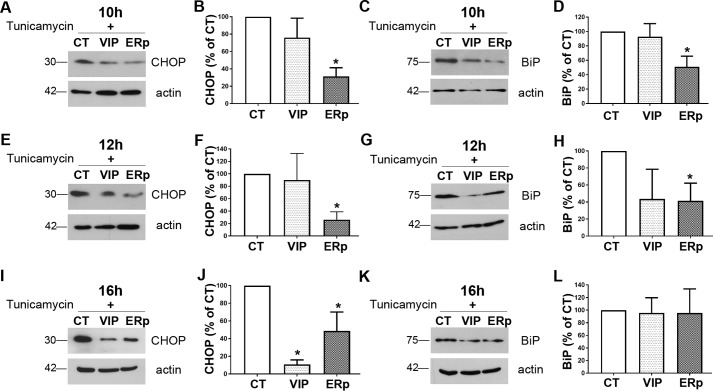
**ERp57 overexpression overcomes ER stress in cells.** N2a cells transiently transfected with ERp57 (*ERp*) or VIP36 (*VIP*) or cells transfected with a control plasmid (*CT*) were treated with tunicamycin (2.5 μg/ml) 48 h after transfection to induce ER stress in the cells. Cells were lysed 10, 12, and 16 h after starting tunicamycin treatment, and cell lysates were analyzed by immunoblotting for the ER stress markers BiP and CHOP. *A*, *B*, *E*, *F*, *I*, and *J*, representative immunoblots showing the amounts of CHOP expressed upon ER stress induction in cells and densitometric analysis represented as percentage of control at 10 h (*A* and *B*), 12 h (*E* and *F*), and 16 h (*I* and *J*). Actin was used as a loading control. *C*, *D*, *G*, *H*, *K*, and *L*, representative immunoblots showing the amounts of BiP expressed upon ER stress induction in cells and densitometric analysis represented as percentage of control at 10 h (*C* and *D*), 12 h (*G* and *H*), and 16 h (*K* and *L*) (*n* = 3–4). *, *p* ≤ 0.05.

Furthermore, we looked into ER stress markers in overexpressing cells after inducing ER stress to delineate whether overexpression of ERp57 and VIP36 has any effect on ER stress. As before, we transfected N2a cells with ERp57, VIP36, or control plasmids. After 48 h of transfection, the cells were treated with tunicamycin. The cells were then lysed 10, 12, and 16 h after treatment with tunicamycin. Immunoblotting was done for ER stress markers such as BiP and CHOP (Fig. 7). ERp57-overexpressing cells significantly overcame ER stress induced in the cells compared with control cells, as evidenced by expression of lower levels of the ER stress markers CHOP and BiP (Fig. 7). We found a statistically significant reduction of BiP in ERp57-overexpressing cells 10 and 12 h after treatment but not at 16 h. ER stress–mediated CHOP expression was delayed in ERp57-overexpressing cells at all three time points (10, 12, and 16 h) compared with control cells (Fig. 7, *A*, *B*, *E*, *F*, *I*, and *J*). VIP36 overexpression did not significantly lower expression of the ER stress markers BiP and CHOP compared with control cells both 10 and 12 h after treatment; however, it lowered CHOP expression 16 h after treatment.

Taken together, these data demonstrate that overexpression of quality control proteins like ERp57 and VIP36 has the potential to reduce the accumulation of PrP aggregates in ER stress situations. Furthermore, ER stress itself is diminished. Consequently, the pool of PrP^C^ amenable for cellular prion conversion is reduced, providing a mechanistic explanation for observing less prion propagation in such cells.

### Application of lentiviruses expressing ERp57 prolongs the survival time of prion-infected mice

**Figure 8.**
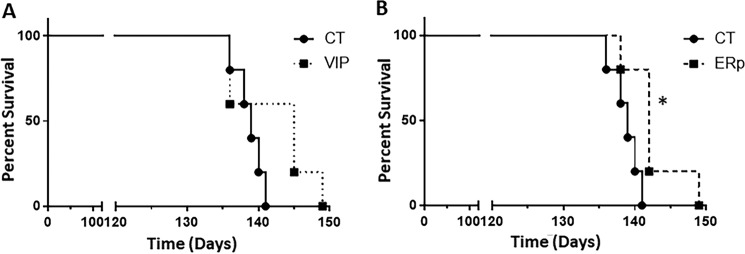
**Application of ERp57-expressing lentiviruses increases survival in prion-infected mice.** Mice were inoculated intracerebrally with 1% brain homogenate from terminally sick mice infected with 22L prions, and 50 day after prion inoculation, mice were given ERp57-expressing (*ERp*, *n* = 5) or VIP36-expressing (*VIP*, *n* = 5) lentiviruses intracerebrally. Control mice (*n* = 5) were inoculated with mock virus. The animals were monitored for clinical prion disease, and the survival of each animal was recorded. *A*, percent survival of control mice (*CT*) and VIP36 lentivirus–inoculated mice. *B*, percent survival of control mice and ERp57 lentivirus–inoculated mice. *, *p* ≤ 0.05.

To test whether the proof of concept obtained in cultured cells can be validated *in vivo*, we applied recombinant lentiviruses expressing ERp57 or VIP36 into the brains of prion-infected mice. Five FVB mice per group were infected intracerebrally with prions. One group received an intracerebrally ERp57-expressing virus, one received a virus expressing VIP36, and the control group received mock virus, all 50 days after prion inoculation. ERp57-expressing virus–inoculated mice showed statistically significant longer incubation times compared with controls (*p* = 0.0206; ERp57 *versus* control group mean survival, 142.6 ± 1.8 *versus* 138.8 ± 0.9), as shown in Fig. 8. Mice treated with a lentivirus expressing VIP36 did not show a statistically significant difference in survival compared with controls. Taken together, these data show that application of lentiviruses expressing ERp57 has the potential to prolong the incubation time to clinical prion disease in prion-infected mice, corroborating our data *in vivo*.

## Discussion

Protein misfolding diseases such as prion disease, Alzheimer's disease, and Parkinson's disease have been associated with ER stress and activation of the UPR in their pathogenesis (25, 33–36). The accumulation of misfolded proteins, as typical for these diseases, and calcium perturbation have been shown to be potential causes for induction of acute and chronic ER stress (26, 37). Chronic ER stress in cells has been linked to up-regulation of signaling pathways that lead to apoptosis and result in cell death and neurodegeneration (38, 39). Several studies, including ours, have shown that ER stress, expression of selected ER quality control molecules, and prion propagation can be interconnected (24, 27, 40). We have demonstrated previously that detergent-insoluble PrP aggregates accumulate in noninfected and infected cells upon ER stress induction, which can lead to increased PrP^Sc^ levels in prion-infected cells (24). Under such conditions, PrP aggregates appear more prone to prion conversion, as demonstrated by another group recently utilizing protein misfolding cyclic amplification (22). Interestingly, our previous work showed that the ER stress–mediated increase in PrP aggregates and in PrP^Sc^ can be reversed by transient overexpression of selected quality control molecules such as ERGIC-53 and EDEM3 (24). Recently, a similar approach has been described for BiP/Grp78, where overexpression of BiP decreased prion propagation, and down-regulation of it resulted in increased PrP^Sc^ levels in cells and shortened incubation periods in prion-infected mouse models (27). Other studies suggested that overexpression of ER chaperones such as BiP/Grp78 and calnexin leads to reduction of β-amyloid peptide, which is involved in the pathogenesis of Alzheimer's disease *in vitro* (41). Moreover, overexpression of the ER chaperone ERp57 in neurons was protective against toxicity induced in cells by addition of exogenous PrP^Sc^ and regulated the expression and maturation of PrP^C^ in cells (28, 29). Additional *in vivo* evidence comes from various studies of prion and other neurodegenerative diseases that show an increase in ER quality control molecules, including ER chaperones and disulfide isomerases, in diseased brains (42–46).

However, the direct role of these chaperones in prion disease is not fully understood. Based on these studies, we hypothesized that stable overexpression of selected quality control molecules will lead to reduced PrP misfolding in the early secretory pathway, which, in turn, will affect the propagation of prions in cells. Here we describe the effects of overexpression of two selected quality control molecules involved in folding (ERp57) and secretory protein cargo transport (VIP36).

### Overexpression of ERp57 has anti-prion effects in persistently infected cells, reduces infection in acutely infected cells, and prolongs incubation time in prion-infected mice

ERp57 is one of the disulfide isomerases that, along with calnexin and calreticulin, help with folding of glycoproteins (47). ERp57 has been found up-regulated in the brain of Creutzfeldt–Jakob disease patients (46). *In vitro*, ERp57 was neuroprotective against PrP^Sc^-mediated cellular toxicity (28). Moreover, axonal regeneration was promoted by ERp57, indicating its potential neuroprotective role (48). Although it has been suggested as a possible therapeutic target before, its role in prion infection and prion propagation has not yet been defined. We wanted to transiently and, more importantly, stably overexpress ERp57 in already chronically prion-infected cells and assess whether this affects the levels of PrP^Sc^. In addition, we wanted to see whether cells with increased expression of ERp57 are less susceptible to prion infection. To stably overexpress an epitope-tagged ERp57 in persistently prion-infected neuronal cells, we used a lentiviral approach. Over several passages, upon transduction, we found that ERp57 was overexpressed. This was correlated with a significant reduction of PrP^Sc^ up to passage 3, corresponding roughly to 15 days. Although this is consistent with previous studies using transient overexpression of quality control proteins such as BiP, ERGIC-53, and EDEM3, we overexpressed such a molecule over longer periods of time in infected cells. This is the first study that addresses whether such prolonged overexpression can interfere with acute prion infection in cultured cells. Our data clearly show that ERp57-overexpressing cells are less susceptible to prion infection, an effect that was still detectable 25 days after infection.

Besides immunoblot for detection of PK-resistant PrP^Sc^, we used the RT-QuIC assay for prion seeding and conversion and PrP^Sc^-specific confocal microscopy to confirm our results. RT-QuIC is a sensitive *in vitro* technique to detect the ability of PrP^Sc^ seed in prion-infected materials to convert a recombinant PrP^C^ substrate into ThT-positive PrP aggregates in real time (49, 50). Using this technology, prion conversion activity has been detected in brain materials, cerebrospinal fluid, urine, feces, blood, saliva, and other body fluids and tissues in various species (30, 50–52). Moreover, we and others have used RT-QuIC to monitor the anti-prion activity of chemical compounds and anti-PrP antibodies (53, 54). In this study, we compared the seeding activity of infected cell lysates from overexpressing and control cells. To make an easy and meaningful comparison, our analysis is based on one dilution (10^−2^) in RT-QuIC, as shown before by a study where a single dilution was used to validate and optimize the factors for improving the assay (55). In alignment with our immunoblotting and confocal microscopy studies, RT-QuIC data demonstrated a very pronounced reduction of prion seeding activity in ERp57-overexpressing, persistently infected cells compared with control cells. Taken together, our data demonstrate that stable overexpression of ERp57 has the potential to reduce PrP^Sc^ and prion seeding activity in chronically and newly infected cells.

*In vivo*, local application of lentiviruses expressing ERp57 to the brain on day 50 after prion inoculation extended the survival time of prion-infected mice. Lentiviruses expressing RNAi against PrP have been reported to down-regulate PrP expression and to mitigate prion infection (56). The effect obtained in our study was moderate, as the expected area of transgene overexpression is very local because of the nonreplicating nature of the used lentiviral vector. As a system for more widespread overexpression, an adeno-associated virus applied intraventricularly (57) could be more efficient in future studies.

### Molecular mechanisms underlying the ERp57 effects on prion infection

Net changes in cellular prion infection could be due to effects on the amount and localization of PrP^C^, on the interaction of PrP^C^ with PrP^Sc^, or on cellular PrP^Sc^ clearance. Because a direct interaction of ERp57 with PrP^Sc^, the vast majority of which is found in the endocytic pathway, is very unlikely, we favor a direct or indirect effect of ERp57 overexpression on the quality of the PrP^C^ pool eligible for cellular prion conversion. As ERp57 is a quality control protein in the early secretory pathway, its effect on proper folding of PrP^C^ could result in less misfolded or better folded PrP that reaches the cellular locale of prion conversion, as described by us for ER stress situations (24). It has been demonstrated already that PrP^C^ and ERp57 can physically interact, which is not surprising for a glycoprotein and an ER-resident chaperone (29). Other indirect possibilities would be that this results in less PrP^C^ at the plasma membrane or that ER stress caused by prion infection or other signaling pathways is involved. As reported in a previous study, ERp57 can regulate PrP expression *in vitro* and *in vivo* (29). In our system, we also found that ERp57 overexpression, both transient and stable, resulted in higher PrP^C^ levels in the cells. This result clearly specifies that the decrease in PrP^Sc^ in ERp57-overexpressing prion-infected cells was not due to less PrP^C^ being available for conversion. More likely, the pool of PrP^C^ is less favorable for prion conversion. This might be the reason why we found a lowered susceptibility of ERp57-overexpressing, noninfected cells to prion infection.

Our previous studies suggested that ER stress causes deterioration in the quality of PrP^C^, resulting in PrP aggregate formation, followed by enhanced production of PrP^Sc^ in infected cells. We also found that transient overexpression of selected quality control molecules decreased detergent-insoluble PrP aggregates (24). To be able to test the effect of transient and stable overexpression of ERp57 on ER stress, we used treatment with tunicamycin as an experimental model. As a readout, we focused on PrP aggregate formation and induction of typical ER stress marker proteins. These studies showed that ERp57-overexpressing cells significantly overcame ER stress-mediated PrP aggregate formation, resulting in less PrP aggregates in the insoluble pellet fraction compared with control cells. In contrast to a study where ERp57 was found not to affect the ER stress situation in cells (29), our study also suggested that ERp57-overexpressing cells significantly overcame ER stress induced in the cells. This was illustrated by expressing lower levels of the stress markers BiP and CHOP. This discrepancy in the studies might be due to use of different cell lines (mouse embryonic fibroblasts and NSC34 cells in the previous study). In our study, we found that after ER stress induction, CHOP was reduced in VIP36- and ERp57-overexpressing cells 16 h after treatment. Again, this might be due to different cell lines and different approaches used. We measured CHOP at the protein level by immunoblot, whereas the previous study looked into mRNA levels.

In summary, our data strongly indicate that overexpression of ERp57 directly affects the pool of PrP that is eligible for cellular prion conversion, resulting in less prion conversion. This also impacts acute prion infection scenarios, making such cells less susceptible to infection. In addition, ERp57 overexpression improves ER stress in compromised cells, providing additive effects.

### Anti-prion effects of VIP36 overexpression

VIP36 has not been described previously in the context of prion diseases. We found VIP36 as potential target when screening for quality control molecules with anti-prion effects. VIP36 has been shown to have a role in post-ER quality control of human α-1 antitrypsin and recycles between the ER and Golgi (20, 58, 59). We found that stable overexpression of VIP36 in prion-infected cells significantly reduced PrP^Sc^ but not as strongly as ERp57. Interestingly, in acute prion infection, VIP36-overexpressing cells showed a very slight reduction in PrP^Sc^ levels compared with control cells in immunoblot analysis. On the other hand, in RT-QuIC analysis, the seeding activity of VIP36-overexpressing cells was reduced compared with control cells, suggesting that not all PK-resistant PrP^Sc^ correlate with prion conversion and propagation (60–62). Similar to ERp57, VIP36 overexpression decreased detergent-insoluble PrP aggregates upon ER stress induction, suggesting functions of VIP36 in ER stress. However, the effect of VIP36 overexpression on regulation of CHOP in cells compared with control cells was not as prominent as for ERp57 at early time points. However, at 16 h, CHOP was significantly reduced in VIP36-overexpressing cells, suggesting functions of VIP36 in ER stress. We still have to clarify whether PrP^C^ and VIP36 interact directly.

In the transient transfection situation, we found slightly different results regarding cell type and prion strain used. ERp57 overexpression, which reduced PrP^Sc^ under the stable expression condition, had a consistent effect upon repetitive transient transfection in ScN2a-22L cells. In ScMEF cells, ERp57 significantly reduced PrP^Sc^ levels in Me7-infected MEFs but not in RML-infected MEFs, possibly ruling out a generalized effect of overexpression on all prion strains. Similarly, transient VIP36 overexpression significantly decreased prion infection in ScN2a-22L and ScMEF-Me7 cells but not in ScMEF-RML cells. It will be interesting to see whether down-regulation of ERp57 or VIP36 in cells results in elevated levels of PrP^Sc^ and prion conversion activity and whether this would make cells more susceptible to infection.

Taken together, our data suggest that manipulation of ERp57 and VIP36 expression could be a promising target against prion diseases. We show that ERp57 and VIP36 overexpression modulates PrP quality control and PrP^Sc^ propagation in acute and chronic infection. Overexpression directly affects the biochemical properties of PrP and helps cells to cope with ER stress. We are in the process of validating this proof of concept obtained *in vitro* in mouse models infected with prions. Overexpression in the brain will be achieved using recombinant lentiviruses and adeno-associated virus (AAV) delivery systems. This will also allow us to combine VIP36- and ERp57-encoding viruses in the form of combination therapy. Given the many similarities between prion diseases and other human neurodegenerative disorders, our approach may also be of interest for other protein misfolding diseases.

## Experimental procedures

### Reagents and antibodies

PK and Pefabloc (a PK inhibitor) were purchased from Roche and Lipofectamine LTX Plus reagent from Invitrogen. Unless stated otherwise, all other reagents and chemicals were obtained from Sigma-Aldrich. Primary antibodies were purchased as follows: anti-HA (Abcam), anti-myc (EMD Millipore), anti-BiP (Santa Cruz Biotechnology), anti-CHOP (Santa Cruz Biotechnology), and anti-β-actin (Sigma-Aldrich). The anti-PrP mAb 4H11 has been described previously (63). Peroxidase-conjugated secondary antibodies were from Jackson ImmunoResearch Laboratories (goat anti-mouse HRP and goat anti-rabbit HRP).

### Maintenance of cell culture

The mouse neuroblastoma cell line N2a was purchased from the ATCC (CCL-131) and maintained in Opti-MEM Glutamax medium (Gibco) containing 10% fetal bovine serum (Sigma) and penicillin/streptomycin in a 5% CO_2_ atmosphere. MEFs were immortalized fibroblasts obtained from Dr. N. Mizushima (Tokyo Medical and Dental University, Japan). The MEF cell line was maintained in DMEM Glutamax (Gibco) containing 10% fetal bovine serum (Sigma). Human embryonic kidney (HEK) cells for lentiviral transduction were purchased from Invitrogen. The HEK cell line was maintained in DMEM Glutamax (Gibco) containing 10% fetal bovine serum (Sigma).

### Ethics statement

All animal experiments were performed strictly following the Canadian Council for Animal Care guidelines and were approved by the University of Calgary Health Sciences Animal Care Committee. The experiments involving the propagation of 22L prions in C57Bl/6 mice (obtained from Charles River Laboratories) were approved under protocol number AC14-0025. Studies involving prion infection and application of recombinant lentiviruses were approved under protocol number AC13-0215.

### Primary prion infection

The mouse-adapted scrapie strain 22L was used. This prion strain was propagated in C57Bl/6 mice. To prepare brain homogenates, brains were homogenized in PBS at a final concentration of 10% (w/v) and stored at −80 °C. For primary prion infection of cells, 1 × 10^6^ cells were seeded in a 6-cm culture dish. After 24 h, the culture medium was removed, and cells were overlaid with 1% brain homogenate in appropriate serum-free culture medium (900 μl). After 5 h of incubation, 1 ml of complete culture medium was added. The medium was removed 24 h later, and cells were washed once with PBS before fresh culture medium was added to the cells. For detection of PrP^Sc^ upon primary prion infection, cells were lysed, and an aliquot of the cell lysate was subjected to PK digestion and immunoblot analysis.

### Transient transfection with plasmid

Lipofectamine LTX was used for all plasmid transfection experiments according to the manufacturer's protocol. Briefly, cells were plated in 6-cm plates for each experiment at a density of 3 × 10^5^. Plasmid and Lipofectamine Plus reagent plus Opti-MEM were mixed and incubated for 5 min, added to LTX reagent and Opti-MEM, and kept for 15 min at room temperature. This solution was added stepwise to cells and gently mixed. Cells were incubated at 37 °C overnight, and the medium was replaced with fresh complete medium. After 48 h, a second transfection was done. 96 h after the first transfection, cells were lysed and processed for immunoblot analysis. The following plasmids have been used in this study: pCDH-CMV-MCS-puro-TA-GFP (Clontech), vector control), pCDH-CMV-ERp57-puro-TA-GFP (ERp57)) and pCDH-CMV-VIP36-puro-TA-GFP (VIP36).

### Lentiviral transduction of cells

For stable overexpression of ERp57 and VIP36, the expression vector pCDH (dual promoter) was purchased from (Clontech). The envelope vector pMD2.G and the packaging vector pPAX for producing lentiviral particles were purchased from Addgene. For production of recombinant lentiviruses, HEK293FT cells were co-transfected with a lentiviral envelope vector, lentiviral packaging vector, and either the lentiviral plasmid ERp57 or VIP36 using Lipofectamine LTX transfection reagent according to manufacturer's directions. The medium of HEK293FT cells containing lentiviral particles was filtered (0.45 μm) to remove cellular debris and used for transduction of recipient cells. For lentiviral transduction of N2a/ScN2a-22L cells, 5 × 10^4^ cells were plated in a 12-well culture dish. For N2a cells, cells were incubated the next day with 4 μg/ml Polybrene solution (Sigma) for 30 min before overnight exposure to 1 ml of medium containing lentiviral particles. For ScN2a-22L cells, the virus was directly added without Polybrene treatment. Then, cells were cultivated further in normal culture medium. The efficacy of lentiviral gene transfer was controlled by analyzing GFP expression by fluorescence microscopy followed by puromycin selection.

### PK digestion and immunoblotting

Immunoblot analysis was done as described previously (64). Briefly, confluent cells were lysed in cold lysis buffer (10 mm Tris-HCl (pH 7.5), 100 mm NaCl, 10 mm EDTA, 0.5% Triton X-100, and 0.5% sodium deoxycholate) for 10 min. Aliquots of lysates were incubated with PK for 30 min at 37 °C. A proteinase inhibitor (0.5 mm Pefabloc) was used to stop PK, and samples were directly precipitated with methanol. For samples without PK treatment, Pefabloc was added directly, and samples were precipitated with methanol. Precipitated proteins were resuspended in TNE buffer (50 mm Tris-HCl (pH 7.5), 150 mm NaCl, and 5 mm EDTA). Samples were run on 12.5% SDS-PAGE (10.5% gel for ER stress markers), electroblotted on Hybond P 0.45 polyvinylidene difluoride membranes (Amersham Biosciences), and analyzed by immunoblot using Luminata Western chemiluminescent HRP substrates (Millipore). The densitometric analysis of immunoblots was done using ImageJ software.

### RT-QuIC

#### 

##### Preparation of recombinant protein

Recombinant prion protein was prepared as described previously (50). Briefly, mouse PrP (amino acids 23–231) was produced using transformed bacteria cultured in Luria-Bertani (LB) medium using the Overnight Express Autoinduction System (Novagen) to induce protein expression. Inclusion bodies were isolated from pelleted cells using Bug Buster Master Mix (Novagen) and stored at −20 °C. For purification of recombinant PrP, inclusion bodies were solubilized in (8 m guanidine HCl, 100 mm sodium phosphate, and 10 mm Tris-HCl (pH 8.0)) and centrifuged at 16,000 × *g* for 5 min, and then the supernatant was added to preincubated nickel-nitrilotriacetic acid Superflow resin beads (Qiagen) in denaturing buffer (6 m guanidine HCl and 100 mm sodium phosphate (pH 8.0)) for 1 h at room temperature. The beads were then packed into an XK16 glass column (GE Healthcare Life Sciences; length, 200 mm). Using an Amersham Biosciences ÄKTA Explorer FPLC unit running with Unicorn software (version 5, GE Healthcare Life Sciences), protein was refolded by a gradient from 100% denaturing buffer to 100% refolding buffer (100 mm sodium phosphate and 10 mm Tris-HCl (pH 8.0)) over 4 h. The column was washed for 30 min with refolding buffer, and proteins were eluted using a linear gradient from 100% refolding buffer to 100% elution buffer (500 mm imidazole, 100 mm sodium phosphate, and 10 mm Tris-HCl (pH 5.8)). The central portions of the *A*_280_ UV peak were collected into dialysis buffer (10 mm sodium phosphate (pH 5.8)). Purified protein was filtered using a 0.22-μm filter and transferred into a Slide-A-Lyzer dialysis cassette (molecular mass, 10 kDa; Thermo Scientific) for dialysis. The dialyzed protein concentration was measured using the BCA protein assay (Thermo Scientific). The solution was aliquoted and kept at −80 °C until use.

##### RT-QuIC assay

The real-time quaking-induced conversion assay was set up as described previously (51). Briefly, 98 μl of master mix containing 20 mm sodium phosphate (pH 7.4), 300 mm NaCl, 1 mm EDTA, 10 μm Thioflavin T, and 0.1 mg/ml rPrP substrate was added to each well of a black-walled 96-well optical bottom plate (Nalge Nunc International). The seeds were prepared by 10-fold serial dilutions of brain homogenate or cell homogenate in seed dilution buffer. 2 μl of seed in each dilution was added to wells in quadruplicate reactions. Plates were sealed with Nunc amplification tape (Nalge Nunc International) and incubated in a FLUOstar Omega (BMG Labtech, Cary, NC) plate reader for 50 h at 42 °C. The 1 min shaking (700 rpm) and 1 min rest cycle was adopted throughout the incubation. ThT fluorescence measurements (450 nm excitation and 480 nm emission) were taken every 15 min, and the average from four replicate wells was calculated and plotted against reaction time.

### Immunofluorescence

ScN2a cells at 80–90% confluency were fixed in 4% paraformaldehyde for 30 min at room temperature, followed by quenching in 50 mm NH_4_Cl and 20 mm glycine at room temperature for 10 min. Cells were then permeabilized in PBS containing 5% fetal bovine serum and 0.5% Triton X-100 (PBSST) for 30 min before incubation of 6 m guanidine hydrochloride at room temperature for 8 min to denature cellular prions. Cells were incubated with anti-PrP mAb 4H11 at 1:100 diluted into phosphate-buffered saline with Triton X-100 (PBST) overnight at 4 °C. The secondary antibody Cy3 goat anti-mouse (Jackson ImmunoResearch Laboratories) was used at 1:200 to visualize the immunostaining signal. All quantitative images were captured under a ×63 oil lens at the same acquisition settings on a Zeiss LSM 700 confocal microscope. The overall immunofluorescence intensity of PrP^Sc^ from an image was measured with ImageJ software after the background was filtered by the same threshold applied to the same series images. Overall intensity was divided by the number of the cells obtained from the same image as quantified by the ImageJ “analyze particle” command to calculate the averaged immunofluorescence intensity per cell.

### Detergent solubility assay

The detergent solubility assay was done as described previously (24). Briefly, 0.5 mm Pefabloc and *N*-lauryl sarcosine at a final concentration of 1% were added to the postnuclear cell lysates. The solution was centrifuged at 4 °C for 1 h at 100,000 × *g* using a Beckman TL-100 centrifuge. The supernatant fraction was collected and precipitated with methanol, and the pellet was resuspended in sample loading buffer (7% SDS, 30% glycerin, 20% β-mercaptoethanol, and 0.01% bromphenol blue in 90 mm Tris-HCl (pH 6.8)) for immunoblotting. Both the supernatant and pellet fractions were analyzed by SDS-PAGE.

### Mouse bioassay

Five-week-old female FVB mice (obtained from Charles River Laboratories) were inoculated under anesthesia in the right parietal lobe with 20 μl of 1% brain homogenate from terminally sick mice inoculated with prion strain 22L. For the intracerebral inoculation, 25-gauge disposable needles were used. After inoculation, mice were checked daily for any adverse conditions. Fifty days after prion inoculation, the animals were inoculated similarly in the right parietal lobe with 20 μl of recombinant lentiviruses (ERp57 or VIP36) with a titer of 2 × 10^6^ international units/animal. The control group (CT) received mock virus. The animals were monitored daily for progression of prion infection after the clinical signs started in animals. At the terminal stage of disease, animals were anesthetized, followed by euthanasia using CO_2_ overdosing. The survival time of each animal was recorded.

### Statistical analysis

Statistical analysis was performed using GraphPad Prism (GraphPad Software, version 7.03) using nonparametric Mann–Whitney test for pairwise comparisons between control and treated groups. Statistical significance for all immunoblots was expressed as mean ± S.E. For survival, the percent survival was plotted in a Kaplan–Meier plot, and a log-rank (Mantel–Cox) test was performed for statistically significant difference between groups: *, *p* ≤ 0.05; **, *p* ≤ 0.01; ***, *p* ≤ 0.001.

## Author contributions

S. T., M. B. A., and H. M. S. conceptualization; S. T., D. H. A., and H. M. S. data curation; S. T. software; S. T., B. A., D. H. A., L. L., M. B. A., and H. M. S. formal analysis; S. T., B. A., D. H. A., and H. M. S. validation; S. T., B. A., D. H. A., L. L., and M. B. A. investigation; S. T. visualization; S. T., B. A., D. H. A., L. L., M. B. A., and H. M. S. methodology; S. T. and H. M. S. writing-original draft; S. T., B. A., D. H. A., L. L., M. B. A., and H. M. S. writing-review and editing; H. M. S. resources; H. M. S. supervision; H. M. S. funding acquisition; H. M. S. project administration.

## Footnotes

2 The abbreviations used are:

PK: proteinase K
ER: endoplasmic reticulum
UPR: unfolded protein response
HA: hemagglutinin
RT-QuIC: real-time quaking-induced conversion
MEF: mouse embryonic fibroblast
HEK: human embryonic kidney
ThT: Thioflavin T.
